# A Probabilistic Atlas of the Pineal Gland in the Standard Space

**DOI:** 10.3389/fninf.2021.554229

**Published:** 2021-05-17

**Authors:** Foroogh Razavi, Samira Raminfard, Hadis Kalantar Hormozi, Minoo Sisakhti, Seyed Amir Hossein Batouli

**Affiliations:** ^1^Neuroimaging and Analysis Group, Research Center for Molecular and Cellular Imaging, Tehran University of Medical Sciences, Tehran, Iran; ^2^Department of Cognitive Psychology, Institute for Cognitive Sciences Studies, Tehran, Iran; ^3^Department of Neuroscience and Addiction Studies, School of Advanced Technologies in Medicine, Tehran University of Medical Sciences, Tehran, Iran

**Keywords:** pineal gland, probabilistic atlas, MRI, normal template, MNI space

## Abstract

Pineal gland (PG) is a structure located in the midline of the brain, and is considered as a main part of the epithalamus. There are numerous reports on the facilitatory role of this area for brain function; hormone secretion and its role in sleep cycle are the major reports. However, reports are rarely available on the direct role of this structure in brain cognition and in information processing. A suggestion for the limited number of such studies is the lack of a standard atlas for the PG; none of the available MRI templates and atlases has provided parcellations for this structure. In this study, we used the three-dimensional (3D) T1-weighted MRI data of 152 healthy young volunteers, and provided a probabilistic map of the PG in the standard Montreal Neurologic Institute (MNI) space. The methods included collecting the data using a 64-channel head coil on a 3-Tesla Prisma MRI Scanner, manual delineation of the PG by two experts, and robust template and atlas construction algorithms. This atlas is freely accessible, and we hope importing this atlas in the well-known neuroimaging software packages would help to identify other probable roles of the PG in brain function. It could also be used to study pineal cysts, for volumetric analyses, and to test any associations between the cognitive abilities of the human and the structure of the PG.

## Introduction

Standard brain MRI templates are used for spatially transforming multiple data to a common coordinate space (Huang et al., 2010), and the use of these templates for such purposes is superior to the traditional methods such as identifying anatomical landmarks (Naidich et al., 2001), or to match cortical surface features (Lancaster et al., 1999). The first common human brain coordinate was the one introduced by Talairach and Tournoux (1988), and currently, the latest Montreal Neurologic Institute (MNI) brain template, known as the ICBM152, is most commonly used in brain imaging studies (Huang et al., 2010).

There are reports that the general features of the brain, such as size, shape, regional volumes, and position of structures, vary across different races and populations due to their different phenotypic, genetic, environmental, and developmental factors (Batouli et al., 2012; Chiang et al., 2012; Sachdev et al., 2013). The MNI templates (Mazziotta et al., 2001) are created by using the data collected from North American individuals (Mandal et al., 2012); a study has illustrated that the brains of Asians and Caucasians are different in shape and size (Bhalerao et al., 2018), suggesting that the analysis of brain images by using a template constructed in a different population may not be appropriate, due to the errors in brain localization (Liang et al., 2015) or imperfect compensations for brain region discrepancies (Argall et al., 2006). Population-specific brain templates improve the accuracy of the studies (Rao et al., 2017), and creating study-specific templates is currently performed in voxel-based morphometric researches (Ashburner and Friston, 2000). Many population-specific brain templates are currently introduced, such as the ones created for infants (Altaye et al., 2008), pediatric population (Wilke et al., 2008), age-specific templates (Rorden et al., 2012), and older adults (Grabner et al., 2006), and also on the Korean (Lee et al., 2005), Chinese (Tang et al., 2010), and Japanese (Quallo et al., 2010) populations.

The MRI has enabled the visualization of the structures of the brain; however, clinical applications or dedicated research needs to localize the brain structures more accurately (Lalys et al., 2010). Brain atlases, which are derived from brain templates (Lalys et al., 2010), are beneficial for the localization and parcellation of brain structures by providing standard anatomical references for them. They are used to reference anatomy in neuroscience, to study the position and shape of the brain structures (Ewert et al., 2018), and to achieve an optimal intensity and spatial resolution (Lalys et al., 2010). The MNI152 template comprehensively covers the neuroanatomy of the brain, with details from the bottom of the cerebellum to the top of the head (Lalys et al., 2010). However, despite the availability of numerous brain atlases, to the best of our knowledge, none of them has provided the anatomical references for the pineal gland (PG), a subcortical brain structure.

The PG is a pinecone-like and small neuroendocrine organ of the brain, and by the secretion of melatonin, it modulates the sleep and circadian rhythms (Atmaca et al., 2019). In humans, this organ reaches full development approximately at the age of 2 years, and although there are reports on the consistency in size and weight of this organ in later life (Nölte et al., 2009), there are reports that its volume is positively correlated with the melatonin level (Nölte et al., 2009). The PG is located in the midline areas of the brain and is connected with the diencephalon through the pineal stalk. The pineal stalk does have an inferior lip, which links the PG to the posterior commissure, and a superior lip that links the PG to the habenular commissure. The pineal stalk possesses a recess at the base of it, which is related with the third ventricle (Reiter, 1981).

The PG is involved in a number of physiological functions, for example in the circadian rhythm (Sun et al., 2009), mood (Al-Holou et al., 2010), sexual maturation and reproduction (Silman et al., 1979), aging (Hasegawa et al., 1987), modulating gonadal activity (Raghuprasad and Manivannan, 2018), and mediating responses to light and altering pigment coloration (Raghuprasad and Manivannan, 2018). There are also reports on the association of PG's function and disorders, such as obesity (Golan et al., 2002), hypertension (Reyes, 1982), bipolar disorder (Sarrazin et al., 2011), and sudden infant death syndrome (Sparks and Hunsaker, 1988). In addition, the PG volume is reported to be lower in patients with insomnia (Bumb et al., 2014), schizophrenia (Findikli et al., 2015), obsessive-compulsive personality disorder (Atmaca et al., 2019), Alzheimer's disease (Matsuoka et al., 2017), attention deficit hyperactivity disorder (ADHD) (Bumb et al., 2016), obesity (Grosshans et al., 2016), and sleep problems (Mahlberg et al., 2009). There are rare reports available in terms of its cognitive roles; a study has hypothesized that the PG has a role in human memory (Batouli and Sisakhti, 2019), which needs further investigation. In addition to the functional roles, this gland is affected by several kinds of lesions, such as the pineocytoma, pineoblastoma, astrocytoma, ependymoma, subependymoma, glioblastoma, etc., as reviewed previously (Hudgins et al., 1987). The rate of pineal cyst incidence is estimated around 10–11% on routine imaging and 20–40% at autopsy (Pham et al., 2015).

The pineal cysts are usually small, asymptomatic, and unilocular, and do not show changes in size (Gokce and Beyhan, 2018), and therefore precisely identifying PG on brain images is essential for pathology purposes (Acer et al., 2012). In addition, the lack of reports on the cognitive roles of this structure could be due to the lack of knowledge on the anatomical landmarks of the PG on the available brain atlases. As a result, this study aimed to provide a standard atlas of the PG in the MNI space, and in the space of a local brain template. This atlas has both clinical and cognitive research applications. Previous studies have also developed atlases for specific structures of the brain, including the basal ganglia and thalamus (Iglesias et al., 2018), and the cerebellum (Park et al., 2014), and it is illustrated that brain atlas are necessary for MRI-based studies of brain structures (Iglesias et al., 2018). We hope that this publicly available atlas could be implemented in the well-known brain imaging data analysis software packages, such as the FSL (FMRIB Software Library, Oxford, UK) and SPM (Statistical Parametric Mapping, UCL, London, UK), and be an aid to enhance our understanding of this brain structure and its roles.

## Methods and Results

### Participants

The data used in this study were from the two previous works (Batouli and Saba, 2020; Batouli et al., 2021). In total, the MRI data from 152 (53F) healthy young individuals were used (in accordance with the number of participants used for building the ICBM152 template). Building a brain template is preferred to be on the data from young and healthy individuals (Huang et al., 2010). They were all right-handed [based on the Edinburgh Inventory (Oldfield, 1971)], mentally and physically healthy (assessed by a general practitioner, MD, PhD in Neuroscience), in the age range of 20–35 years old, and from the same nationality, with at least 14 years of education. Each participant was informed about the general aims of the study, declared his/her assent during the initial interview, and signed the consent form on the test day. The ethics approval was provided for both studies, with the approval numbers of IR.NIMAD.REC.1396.319 and IR.IUMS.REC.1395.899.

The criteria for selection of the individuals were based on the Iranian Brain Imaging Database (Batouli et al., 2021); in summary it included: weight not over 110 kg; not using drugs or alcohol; a consent to participate in all steps of the study; and not being claustrophobic. The following exclusion criteria were also checked by the general practitioner (HK): any diagnosed internal/neurologic disease; long term or current use of medications; any history of chronic headache, tinnitus, dizziness, seizure, or nausea; family history of any disease; any surgery with anesthesia; history of losing consciousness or head trauma; and any metal objects in the body, such as a pacemaker, dental brace, coronary stent, implant, or tattoo. Each participant was also examined by the physician for blood pressure, heart, and respiratory rates, and a neurological examination including vision and hearing. To assess mental health, the Depression Anxiety Stress Scales (DASS-21) (Henry and Crawford, 2005) was administered, normalized for the Persian language and population (Sahebi et al., 2005), and the included participants were in the normal range.

### MRI Imaging

Both studies used similar MRI scanners and protocols. The MRI machine was a Siemens 3 Tesla scanner (Prisma; Siemens Healthcare GmbH, Federal Republic of Germany; Production: 2016), devoted to research at the National Brain Mapping Lab (www.nbml.ir). Using a 64-channel head coil, three-dimensional (3D) T1-weighted anatomical scan (non-contrast) was acquired by using a gradient echo pulse sequence [acquisition time (TA) = 4:12 min; repetition time (TR) = 1,800 ms; echo time (TE) = 3.53 ms; TI = 1,100 ms; flip angle = 7°; voxel size = 1.0 mm × 1.0 mm × 1.0 mm; matrix size = 256 × 256 × 160; average = 1]. Other MRI protocols were also performed in the original works, but the current study only used their T1-weighted data.

### Pineal Parcellation

**Figure 1:**
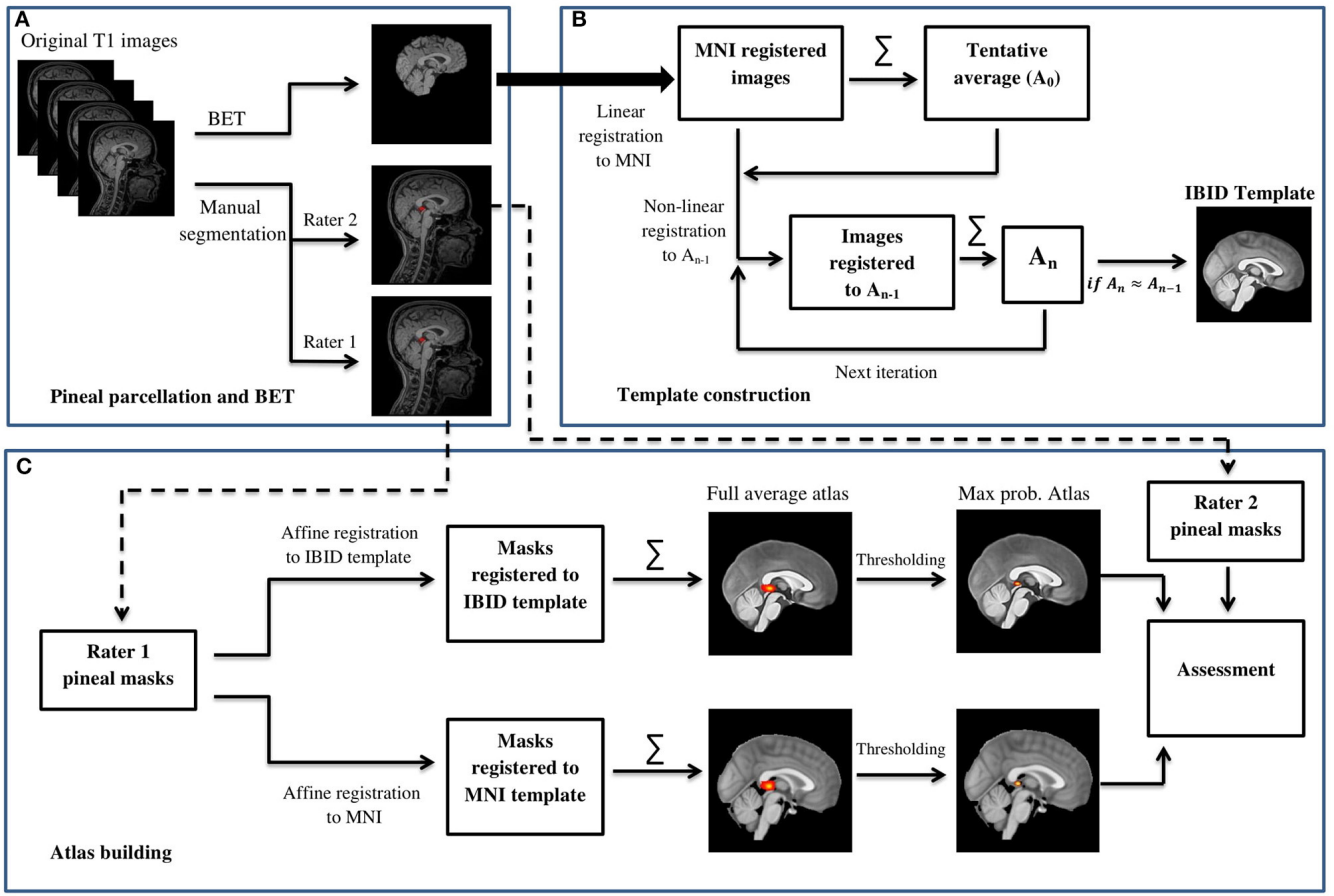
The general flowchart of building the IBID brain template and the pineal gland (PG) atlas. **(A)** Pineal parcellation and BET. **(B)** Template construction. **(C)** Atlas building.

The general flowchart of the methods of this study is provided in Figure 1. The first step was to manually delineate the PG on the MRI scans, which were performed by two experts (an anatomist with a PhD in neuroscience: SR and a medical doctor: HK). The PG is an outgrowth from the diencephalon, which is named as epithalamus, and is attached to it by the pineal stalk. PG is a midline structure of the brain, located at the level of the superior colliculus, inferior to the splenium of the corpus callosum, and posterior to the third ventricle, where it is separated by the great cerebral vein of Gallen (Standring, 2015). This structure is mostly enclosed by cerebrospinal fluid (CSF) except the area to attach to the habenula, and therefore its borders are distinguishable in anatomical images (Bumb et al., 2014).

**Figure 2:**
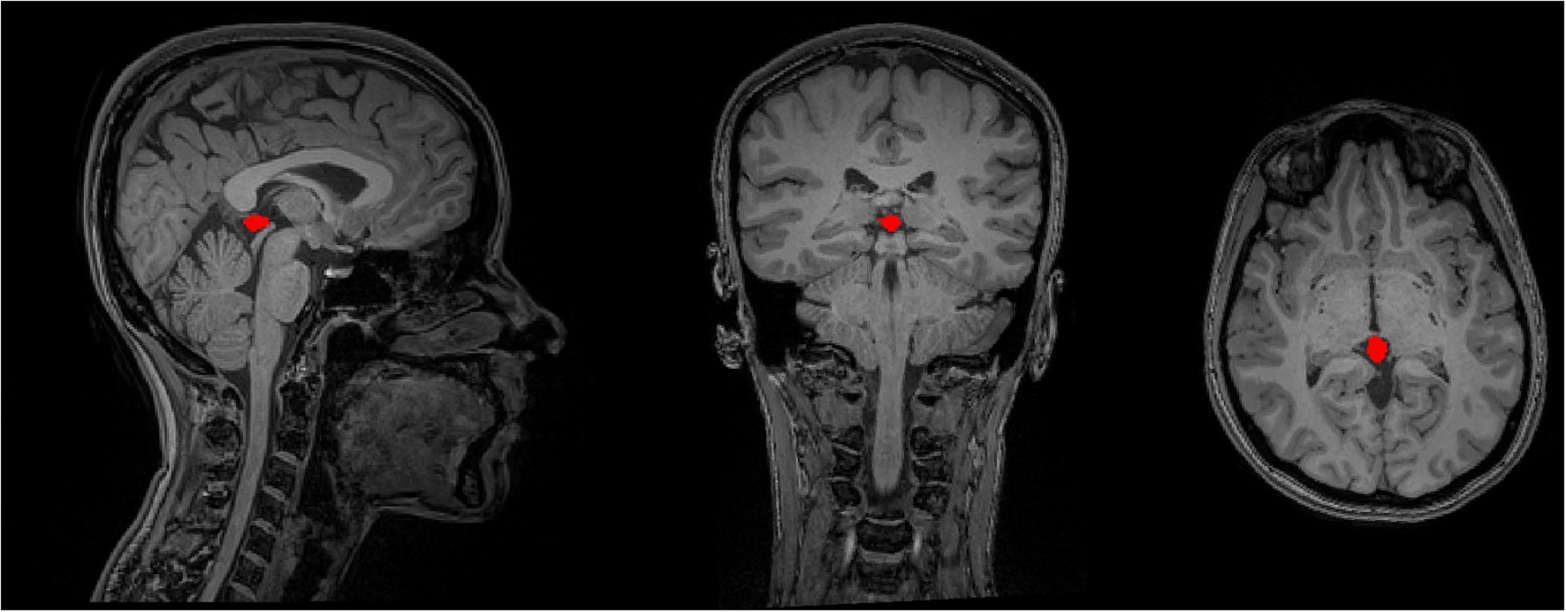
The delineation of the PG's contour on a sample T1-weighted MRI data in the sagittal, coronal, and axial image views.

The contour of the PG was manually delineated by using both transverse and sagittal views of the 3D T1-weighted images, using the ITK-SNAP software (version 3.4.0, www.itksnap.org). First, the bulk of the PG tissue was identified on the transverse plane. For this, axial images were scrolled to the level of the superior colliculi (tectal plate) until the cone-shaped PG and its connection to the habenula in the medial border of the thalamus were identified. The first region of interest (ROI) was drawn here, and the rest of the gland was contoured by scrolling the slices up or down for two or three more slices. The final ROI was rechecked on the sagittal images to separate between the adjacent superior vessels and the PG (Sun et al., 2009). In cases where the PG was cystic, the cyst was also included in the ROI, so that the whole PG volume is selected. Figure 2 illustrates an example of the ROI delineated for the PG.

### Parcellation Reliability

After the PG ROIs were provided by both raters for all the MRI data, we estimated the Intraclass Correlation Coefficient (ICC) between their results, to assess the inter-rater reliability. Based on the ICC estimation guideline suggested previously (Koo and Li, 2016), we chose the ICC variant that measured the absolute agreement under a two-way random ANOVA model. The ICC estimates and their 95% CIs were calculated in MATLAB (version 9.0). The ICCs showed a good agreement between the two raters' PG segmentations, with an average ICC of 0.83 (0.818 < CI <0.841). However, five of the ICCs were outliers (±3 SD from the mean value), and therefore those data were excluded from the study, resulting in a sample size of 147. The ROIs from rater1 were used for the atlas preparation, and the results of rater2 were used for assessing the accuracy of the outcome atlas.

### Brain Extraction

The presence of non-brain tissues in MRI images is considered as a major obstacle for automatic brain analysis techniques such as registration. Thus, we used the FSL Brain Extraction Tool (BET) (Smith, 2002) to remove the non-brain tissues from the T1-weighted images (using the “bias field and neck cleanup” option in BET, with a fractional intensity threshold of 0.35). All the brain-extracted MRI data were checked visually to identify errors such as the residual non-brain tissue or the removed brain tissue, using FSLeyes toolbox; similarity of the results to the standard brain template (MNI152_T1_1mm_brain) was also checked. Eight images did not show a proper brain extraction, which were manually corrected.

### Template Construction

Before the construction of the PG atlas, a local brain MRI template was constructed by using an iterative routine similar to those explained in the previous works (Fonov et al., 2011; Sanchez et al., 2012; Bhalerao et al., 2018). Brain-extracted magnetic resonance (MR) images were the first affine registered to the standard MNI152_1mm_brain template (Mazziotta et al., 2001), using SPM12 coregisteration toolbox (Estimate and Reslice). Then, Linux bash scripting was used to develop an automated pipeline to perform the iterative procedure that follows: (1) all the affine-registered images were averaged to create a tentative average (*A*_0_); (2) each subject's MR image was then registered non-linearly to this tentative average template (*A*_0_); (3) the registered images were then averaged to create a reference template for the next iteration (*A*_n_); and (4) the registration and averaging processes (steps 2 and 3) were applied iteratively to construct the final template.

**Figure 3:**
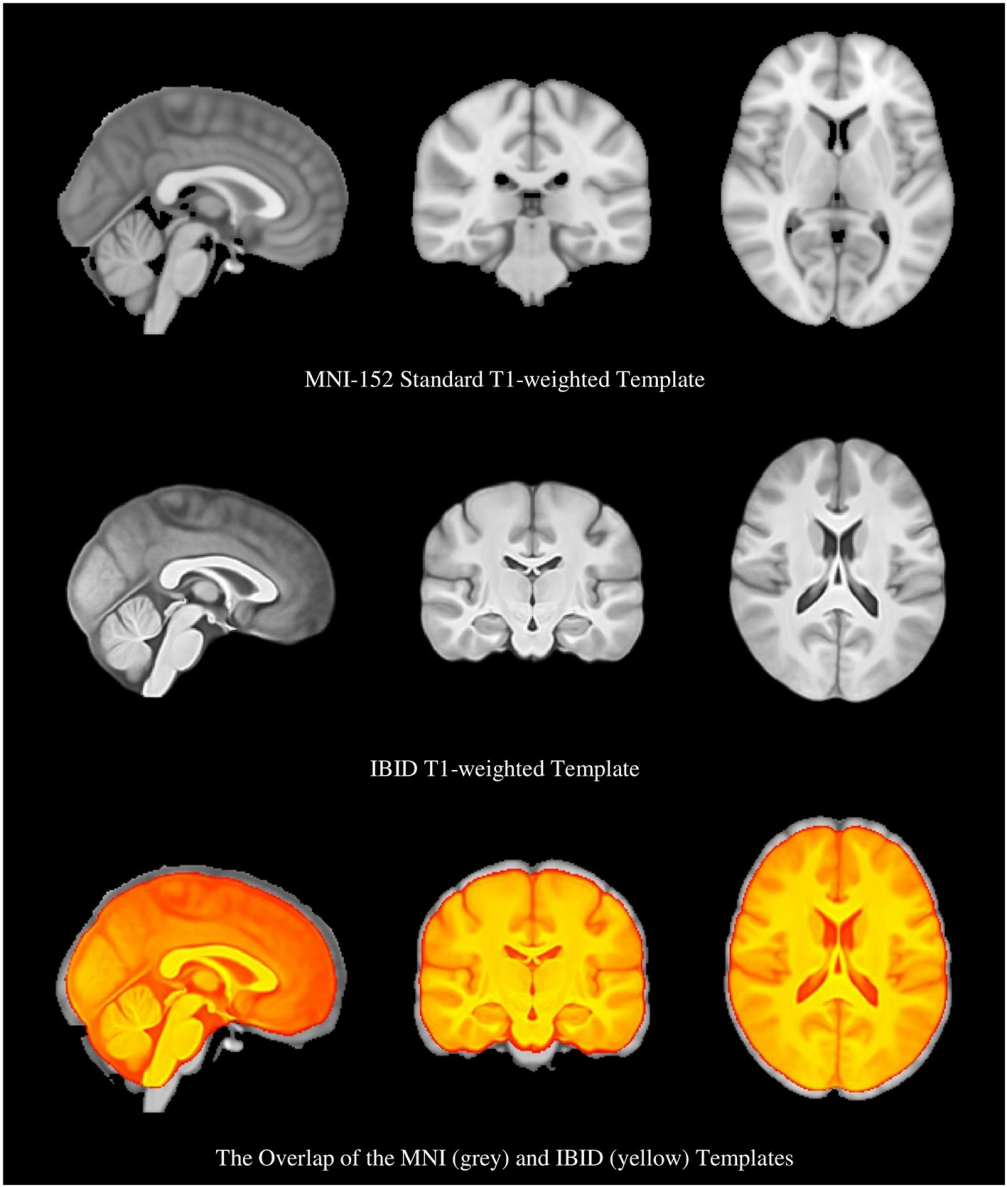
The Montreal Neurologic Institute (MNI) brain template, the local brain template, and the overlap of the two templates, which show their different sizes.

We used Ants Multivariate Template Construction script (Avants et al., 2011) for the steps listed above. Maximum iterations for each pair-wise registration were set to 50 × 50 × 50, based on previous reports (Bhalerao et al., 2018); it means that a maximum value of 50 iterations was performed consecutively at the coarsest resolution (shrink factor 8), middle resolution (shrink factor 4), and fine resolution (shrink factor 1). We used the ANTS symmetric normalization (SyN) transformation model for the affine and diffeomorphic transformations; mean square difference as a similarity metric; and Gaussian regularizer for regularization. The script calculated the root mean square error (RMSE) between the successive average reference templates [e.g., RMSE (A2, A1) or RMSE (A3, A2) and stopped the iterative procedure once leveling of the successive root mean square (RMS) values were obtained, or in other words, the averaged template converged (*A*_n_ ≈ *A*_n−1_)]. The last reference model was then introduced as the final template, which is illustrated in Figure 3.

As it is shown in Figure 3, the IBID template is smaller in size than the standard MNI template. The size of the templates was estimated by two individuals (FR and MS), based on the previously published methods (Rao et al., 2017), and then averaged. It was observed that the anterior commissure and posterior commissure (AC-PC) line is shorter in the IBID template compared to the MNI (26 vs. 28 mm). A similar pattern was also observed in other measures of the template, including length (158 vs. 170 mm), width (130 vs. 138 mm), and height (95 vs. 108 mm).

### Atlas Construction

Two versions of the PG atlas were provided; one by transferring the ROI masks (from rater1) to the local template, and the other by performing the same procedure in the MNI standard space, both accomplished using the SPM12 reslicing toolbox. The PG atlas constructed in the IBID template does have more local applications, whereas the atlas built in the MNI space would be internationally used. The individual ROI masks were transferred to the reference space (the IBID or MNI templates) by reslicing them using the fourth degree b-spline interpolation. The resliced masks were then averaged across participants to form the pineal atlas. The original ROIs were binary, with 1s showing the PG tissue and 0 in other voxels; as a result, a simple averaging resulted in a probability map, which we called the “full average” atlas.

**Figure 4:**
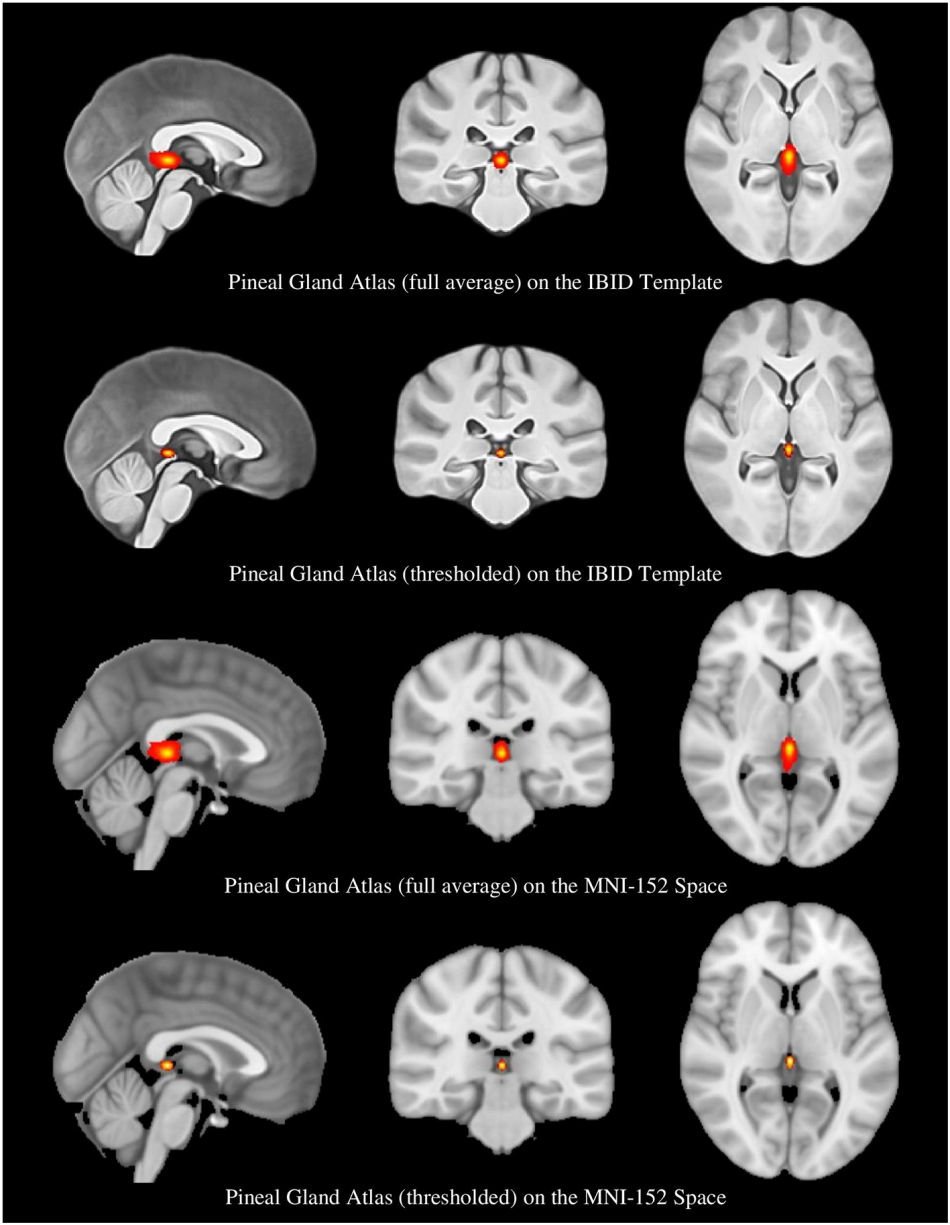
The full average and thresholded PG atlases in the local and MNI spaces.

Another format of the two atlases was also provided, by thresholding the probability maps to remove the voxels with a probability lower than 0.25 (Diedrichsen et al., 2009). This provided a more accurate atlas of the PG, called the “thresholded atlas.” All these four atlases are illustrated in Figure 4.

### Atlas Assessment

We assessed the similarity between the ROIs provided by rater2 with the estimated PG atlas. First, the ROIs (which were in the native space) were registered to the IBID template using SPM12 affine registration. Then, a binary version of the full average atlas in the local space was created; the overlap between each registered ROI and the product atlas was regarded as the similarity index. The results showed a mean overlap of 0.986 ± 0.031 (range: 0.779–1), which showed an appropriate overlap between the estimated PG atlas and the ROIs provided by rater2.

### Template and Atlas Access

The local brain MRI template (called IBID template), as well as the full average and thresholded versions of the PG atlas, in both the local and MNI spaces, are now available to download on our website: http://brainee.ir/pineal-gland-atlas/.

## Discussion

### Summary of the Methods

In this study, first, we provided a local normative brain template by averaging MRI data from 152 healthy young participants. Next, an atlas for the PG was built in both the MNI and the local template spaces. We selected 152 participants in accordance with the number of participants used for building the MNI152 template. A Chinese brain template was based on 56 subjects (Tang et al., 2010); although there is no accurate estimation of the number of data needed to construct an optimal brain template, we trusted the methodology used for building the MNI template. All participants were strictly checked for mental and physical health, as stated previously (Batouli et al., 2021), and a 3T Prisma scanner was used, which is currently one of the best MRI machines. It is illustrated that the signal-to-noise ratio (SNR) is significantly higher at 3 Tesla, compared to 1.5 T MRI (Yongbi et al., 2002; Hoenig et al., 2005). Two individuals with enough expertise in brain anatomy parcellated the PG based on the published standards, and their parcellation results showed a good agreement. Building the template and atlas was also based on robust methods, and the final assessment showed an appropriate precision for the product atlas.

### Brain Templates

Brain templates advance our knowledge of the function, development, and structure of the human brain (Xie et al., 2015), and are helpful for spatial normalization purposes (Altaye et al., 2008). A digital multi-subject template, built from similar acquisitions from several people, captures intersubject variability and provides a common space (Lalys et al., 2010), and the averaging of multiple data improves the quality of the final template by enhancing the SNR and contrast (Lalys et al., 2010).

Our results showed the size of our template to be smaller than the ICBM152 atlas; it had a shorter AC-PC line (26 vs. 28 mm), and also length (158 vs. 170), width (130 vs. 138), and height (95 vs. 108). Although the general size and shape of the brain do not provide detailed information about the brain, these measures are useful for comparison purposes. Many reports are available on the structural differences between the brains of Asians with other populations: a study showed that that the mean width, length, AC-PC distance, and height of the brain is different between Caucasian and Chinese individuals (Tang et al., 2010); the Chinese brain templates were shorter than US templates (Xie et al., 2015); a standard adult Korean brain template was 9% shorter in height, 10% shorter in length, and 1% wider, in comparison to the ICBM-152 brain template (Lee et al., 2005); a significant difference in the shape and size of the Indians' brain template and the ICBM-152 was observed (Rao et al., 2017); and differences were observed in a Japanese template compared to the MNI (Quallo et al., 2010).

Nationality-inappropriate brain templates are not optimal reference MRIs for processing of the MR images of another population (Xie et al., 2015). It also affects the analyses that rely on the spatial registration of individuals' MRI data to the average templates (Xie et al., 2015). A larger size of the MNI template may lead to incorrect deformation and therefore increase registration errors during an analysis (Bhalerao et al., 2018). However, the population-specific brain templates increase the accuracy of results by decreasing the error rates of type I and type II, and enhancing the statistical power (Tang et al., 2010); functional MRI data analysis also benefits from a local template as the accuracy of localization of brain regions improves (Tang et al., 2010).

### Brain Atlases

A probabilistic brain atlas based on the data from multiple individuals can serve as a valid reference for brain localizations (Diedrichsen et al., 2009). For example, segmentation of a few of the brain structures such as the hippocampus is difficult due to reasons such as the intensity of its boundaries with the adjacent brain structures being indistinct. Template-based approaches for segmentation is a help, because a template coordinate system, as the prior knowledge, is used to automatically delineate a structure. Therefore, by using the combination of a template and an atlas that exactly correspond to each other, a more accurate segmentation happens (Ewert et al., 2018).

For the construction of probabilistic atlases, the methods used for the normalization or registration of the individuals' data to a reference template should be precisely selected. As explained in “Methods,” we selected robust algorithms for this section. The spatial resolution of the normalization, the preprocessing of the anatomical data, and the choice of the reference template all show associations with the spatial variances of structures in the template space. Different methods of normalization also lead to variations in the location of structures in the reference space, even if a similar reference template is used. Therefore, it seems essential that the normalization method used for the data analysis matches with the normalization algorithm used to generate the atlas (Diedrichsen et al., 2009). We also thresholded our atlas with an intensity of 0.25. It is reported that the maximum probability maps derived from an atlas can be used to define ROIs in neuroanatomical research (Diedrichsen et al., 2009), and our choice of 0.25 for the threshold was based on a prior work, which developed an atlas for the cerebellum (Schmahmann et al., 1999; Diedrichsen, 2006).

### The Pineal Gland

The mean weight of the PG in adults is between 50 and 150 mg (Hasegawa et al., 1987), and it has been stated that the size of the PG grows from birth until 2 years of age, and then it remains constant until 20 years of age (Sumida et al., 1996). Also, significant correlations are observed between the PG volume and head circumference, body height and body weight (Sun et al., 2009). Increment of the weight of the PG from puberty to older ages was illustrated previously (Tapp and Huxley, 1972), and at the same time, another study showed a negative correlation of the PG volume and age (Bumb et al., 2013), which may be related to more insomnia reports in older adults (Bumb et al., 2014). Another study showed that high consumption of coffee during the lifetime may reduce PG volume, which also impairs the quality of sleep in the elderly (Park et al., 2018). Also, the volume of this structure has shown associations with other brain structures, such as the left parietal lobe and bilateral anterior temporal lobes (Axelrod et al., 1965). The decline in the volume of this structure also shows correlations with cognitive decline (Matsuoka et al., 2017).

Despite the importance of estimating the volume of this structure, as the PG is located in the deep brain and in the complicated pineal region of varying morphological characteristics and shape, the estimation of the PG volume using old methods, such as the ones based on one-dimensional or two-dimensional parameters (e.g., pineal length), is difficult (Acer et al., 2012). The observed variations across studies in the PG volume may also be due to the heterogeneous structure of it, which makes the distinction between the PG and its adjacent brain structures, such as the internal cerebral veins, difficult. As a result, the methods, which yielded a larger volume, may have included some of these adjacent structures in their estimations, or the methods with smaller volumes may have inappropriately excluded relevant structures. In comparing the PG volume across studies, the effect of their different methods cannot be excluded (Sigurdardottir et al., 2016). Using atlases for the estimation of regional brain volumes is common (Batouli et al., 2014; Batouli and Saba, 2020), and therefore our PG atlas could be a help to reduce the discrepancies when estimating the PG volume.

### Limitations

Despite the strengths, the study has a few limitations, which could be suggestions for future works. First is related to having an unequal number of genders in building the template; neither the MNI template nor our brain template included an equal number of male and female MRI data in the construction of the template, which could be influential on the outcome template. Providing templates with an equal number of genders or gender-specific templates would be a suggestion for the future. Also, this template is only based on the data from young adults; age-specific templates would also be necessary for a population-specific template. Also, we used non-linear methods for spatial normalizations during the template construction, and although there are benefits in this approach, different normalization parameters may be applied to different parts of the brain. Using T2-weighted sequences, or post-contrast T1-weighted scans (which have ethical issues of gadolinium injection into normal participants) would possibly help in better identification of the PG cysts. And finally, the agreement between the outcome PG atlas and the parcellations provided by rater2 was not very high, which could be due to a very small size of the PG; obviously, in larger structures, the percentage of disagreement between two maps is lower. All in all, we hope this atlas could enhance our knowledge of the structure, function, and cognitive roles of the PG in the brain.

## Data Availability Statement

The datasets presented in this study can be found in online repositories. The names of the repository/repositories and accession number(s) can be found at: http://brainee.ir/pineal-gland-atlas/.

## Ethics Statement

The studies involving human participants were reviewed and approved by National Institute for Medical Research Development. The patients/participants provided their written informed consent to participate in this study.

## Author Contributions

FR helps in conceptualization, methodology, software, and writing the original draft. SR helps in validation and methodology. HK helps in validation and resources. MS helps in conceptualization, investigation, and resources. SB helps in conceptualization, methodology, investigation, data curation, writing—original draft, writing—review and editing, and supervision. All authors contributed to the article and approved the submitted version.

## Conflict of Interest

The authors declare that the research was conducted in the absence of any commercial or financial relationships that could be construed as a potential conflict of interest.
